# Importance of reference gene selection for articular cartilage mechanobiology studies

**DOI:** 10.1016/j.joca.2015.11.007

**Published:** 2016-04

**Authors:** A. Al-Sabah, P. Stadnik, S.J. Gilbert, V.C. Duance, E.J. Blain

**Affiliations:** Arthritis Research UK Biomechanics and Bioengineering Centre, Sir Martin Evans Building, School of Biosciences, Cardiff University, Museum Avenue, Cardiff, CF10 3AX, UK

**Keywords:** Articular cartilage, Chondrocytes, Mechanobiology, Quantitative PCR, Reference genes

## Abstract

**Objective:**

Identification of genes differentially expressed in mechano-biological pathways in articular cartilage provides insight into the molecular mechanisms behind initiation and/or progression of osteoarthritis (OA). Quantitative PCR (qPCR) is commonly used to measure gene expression, and is reliant on the use of reference genes for normalisation. Appropriate validation of reference gene stability is imperative for accurate data analysis and interpretation. This study determined *in vitro* reference gene stability in articular cartilage explants and primary chondrocytes subjected to different compressive loads and tensile strain, respectively.

**Design:**

The expression of eight commonly used reference genes (*18s*, *ACTB*, *GAPDH*, *HPRT1*, *PPIA*, *RPL4*, *SDHA* and *YWHAZ*) was determined by qPCR and data compared using four software packages (comparative delta-C_t_ method, geNorm, NormFinder and BestKeeper). Calculation of geometric means of the ranked weightings was carried out using RefFinder.

**Results:**

Appropriate reference gene(s) for normalisation of mechanically-regulated transcript levels in articular cartilage tissue or isolated chondrocytes were dependent on experimental set-up. SDHA, YWHAZ and RPL4 were the most stable genes whilst glyceraldehyde-3-phosphate dehydrogenase (GAPDH), and to a lesser extent Hypoxanthine-guanine phosphoribosyltransferase (HPRT), showed variable expression in response to load, demonstrating their unsuitability in such *in vitro* studies. The effect of using unstable reference genes to normalise the expression of aggrecan (ACAN) and matrix metalloproteinase 3 (MMP3) resulted in inaccurate quantification of these mechano-sensitive genes and erroneous interpretation/conclusions.

**Conclusion:**

This study demonstrates that commonly used ‘reference genes’ may be unsuitable for *in vitro* cartilage chondrocyte mechanobiology studies, reinforcing the principle that careful validation of reference genes is essential prior to each experiment to obtain robust and reproducible qPCR data for analysis/interpretation.

## Introduction

Articular cartilage has unique mechanical and physicochemical properties which are responsible for its load-bearing capabilities and near-frictionless movement; this is essential for dissipating mechanical loads applied to the joint^1^. The mechanical properties of articular cartilage are dependent on the composition, structural organisation and integrity of the tissue's extracellular matrix (ECM), which in turn are dependent on, and regulated by mechanical load. Abnormal mechanical load is a primary risk factor for the development of osteoarthritis (OA). Identification of differential gene expression patterns, either as targets or involved in novel mechano-biological pathways in articular cartilage, could be pivotal in providing new insights into molecular mechanisms behind the initiation and/or progression of OA.

Quantitative PCR (qPCR) is the most utilised mRNA quantification method due to its sensitivity in measuring transcript levels^2^. Historically, most mRNA quantification has been performed using glyceraldehyde-3-phosphate dehydrogenase (GAPDH), 18s or ACTB (β-actin) as the reference gene for analysing the response of articular cartilage to mechanical load. However, their suitability as reference genes is questionable, especially due to their potential regulation in a wide variety of physiological states. Gene expression profiling has become increasingly important in our understanding of biological mechanisms, therefore it is surprising that, to date, only one study has previously been performed to identify the reference gene(s) most suitable for normalisation of transcript levels in articular chondrocytes subjected to load^3^. Of the reference genes assessed (18s, ACTB, GAPDH and β2-microglobulin), 18s was deemed to have the most stable expression under the experimental conditions tested^3^. Most ‘reference’ genes have significant roles in cell survival, and as a consequence are expressed in all cell types. However, this does not eliminate the possibility that their expression levels might be modulated in response to specific stimuli e.g., load. Hence, not all ‘reference’ genes should be considered universally suitable for use as qPCR reference genes^2^. Therefore, there is a necessity to validate expression levels of potential reference genes to ensure stability of expression under the experimental conditions of the study, and to prevent misinterpretation of data.

In 2009, the MIQE guidelines (*M*inimum *I*nformation for Publication of *Q*uantitative Real-Time PCR *E*xperiments) was published describing essential criteria required for publication of qPCR data e.g., information on sample acquisition, RNA quality/integrity, qPCR validation and data analysis^4^. Furthermore, the MIQE guidelines indicated that when selecting the most stable reference gene(s), normalisation against one such gene is generally considered unacceptable, and that no fewer than three reference genes is advisable^5^.

A number of software packages including the comparative delta-C_t_ method^6^, geNorm^7^, NormFinder^8^ and BestKeeper^9^ are commonly used to identify and validate stable expression of appropriate reference genes. In the current study, we utilised these software packages to determine the most stable reference genes in articular cartilage subjected to different loading regimens. Calculations of the geometric means of the ranked weightings obtained from the four software packages, using RefFinder (http://www.leonxie.com/referencegene.php) facilitated the identification of suitable reference genes for this tissue type and experimental design. Comparing the outcomes of these different approaches illustrated the impact that reference gene selection can have on experimental results.

In the present study we determined the stability in expression of eight reference genes in chondrocytes from both articular cartilage explants and isolated primary cells, under the influence of load *in vitro*. The data consistently demonstrated that GAPDH and Hypoxanthine-guanine phosphoribosyltransferase (HPRT) showed the highest variability in expression of all reference genes tested. However, the most appropriate reference gene(s) for normalisation of mechanically-regulated transcript levels in articular cartilage tissue or isolated chondrocytes were dependent on individual experimental set-up, reinforcing the necessity to assess reference gene suitability for each study performed.

## Materials and methods

Reagents were purchased from Sigma (Poole, UK) and were of analytical grade or above. All plasticware was certified DNase and RNase-free. Culture medium consisted of Dulbecco's Modified Eagle's Medium/Hams F12-glutamax™ (DMEM/F12(1:1)-glutamax™; Life Technologies, Paisley, UK) supplemented with 100 μg/ml penicillin, 100 U/ml streptomycin, 50 μg/ml ascorbate-2-phosphate and 1× insulin–transferrin–selenium–ethanolamine (1× ITS-X) to maintain the chondrocyte phenotype^10^.

### Cartilage explant and chondrocyte preparation

Full depth articular cartilage explants (5 mm diameter) were taken using a biopsy punch (Selles Medical Limited, Hull, UK) from the *metacarpophalyngeal* joint of 7-day old bovine calves within 6 h of slaughter^11^. Cartilage explants were stabilised in culture medium for 3 days prior to mechanical load. Primary chondrocytes were isolated from full depth articular cartilage slivers from the same tissue and subjected to an enzymatic digestion as previously described^12^; ethical approval was not required for bovine tissue collection. Chondrocytes were plated at high density (4 × 10^6^ cells per well) in 6-well, flat-bottomed pronectin-coated plates (Bio-Flex culture plates; Dunn Laborotechnik, Asbach, Germany). Following isolation, cells were stabilised for 48 h prior to mechanical stimulation. All cultures were maintained at 37°C, 5% CO_2_, 20% O_2_.

### Application of mechanical load

Cartilage explants, immersed in culture media, were subjected to a range of loading regimes (2.5 MPa, 5 MPa or 8 MPa at 1 or 4 Hz, 15 min) using the ElectroForce^®^ 3200 (TA Instruments, Delaware, USA), and gene expression either analysed directly post-cessation of load or 24 h post-load. Chondrocytes were subjected to a physiological tensile strain (7.5% elongation, 1 Hz) for 30 min using the Flexcell FX-3000 system (Flexcell International Corp, Hillsborough, NC, USA)^12^, ^13^, ^14^, and cells processed four hours post-cessation of load to analyse gene expression. Duplicate cultures of explants or cells, devoid of mechanical stimulation, were set up as controls. Cartilage explants were snap frozen and remained in liquid nitrogen until the RNA extraction. Isolated chondrocytes were lysed directly in TRIzol^®^ (1 ml per well) and stored at −80°C until processed for RNA extraction.

### RNA extraction and cDNA synthesis

Cartilage explants were homogenised in TRIzol^®^ (1 ml per 50 mg wet weight tissue: Invitrogen, Paisley, UK) in liquid nitrogen using a dismembrator (Sartorious, Epsom, UK), and RNA extracted as previously described^15^, except for the purification step which was completed using an RNeasy mini kit (Qiagen, Manchester, UK) according to manufacturer's instructions. RNA integrity was assessed using the 2100 Bioanalyzer (Agilent Technologies, Stockport, UK) and RIN scores >8.5 were observed. Complementary DNA (20 μl total volume) was generated from 300 ng total RNA using SuperScript^®^ III reverse transcriptase (Invitrogen, Paisley, UK) and 0.5 μg random primers (Promega, Southampton, UK) according to manufacturer's instructions, and 1 μl utilised in each qPCR assay.

### qPCR analysis

Real-time PCR (polymerase chain reaction) was performed using a MxPro3000 QPCR system (Agilent Technologies, Stockport, UK). A real-time qPCR assay based on SYBR green detection, using Brilliant III Ultra-Fast SYBR^®^ QPCR mix (Agilent Technologies, Stockport, UK) was used for the transcriptional profiling of eight reference genes including 18s^16^, GAPDH^17^, ACTB, HPRT, SDHA (Succinate dehydrogenase complex, subunit A), RPL4 (Ribosomal Protein L4), PPIA and YWHAZ (tyrosine 3-monooxygenase/tryptophan 5-monooxygenase activation protein)^18^. The choice of selected reference gene targets for analysis was largely based on reference gene suitability previously analysed in loaded, isolated chondrocytes^3^, reported to be stable under mechanical perturbation in other tissues^19^, ^20^ or reported to be stable in chondrocytes under other experimental conditions^21^. In addition, the analysis of two commonly examined cartilage genes matrix metalloproteinase 3 (MMP3)^22^ and aggrecan (ACAN)^17^ (Table I) were performed. Both the in-house primers and those primer sequences taken from the literature all span intron-exon boundaries. All reactions were carried out at an annealing temperature of 60°C; cycling conditions were: 95°C–3 minutes (1 cycle), 95°C–15 s followed by 60°C–30 s (40 cycles), 95°C–1 minute followed by 60°C–30 s followed by 95°C–30 s (1 cycle). Primers were purchased from MWG-Biotech AG (Ebersberg, Germany), each utilised at a final concentration of 200 nM and validated using a standard curve of five serial dilutions so that all primer efficiencies were between 90 and 110%^23^. Reactions where sterile water replaced template cDNA were used as negative controls to ensure product specificity. For MMP3 and ACAN expression, relative quantification was calculated using the 2^−ΔΔCT^ method^24^, using the unloaded controls as a reference group to quantify relative changes in target gene expression.

### Determination of reference gene expression stability

To identify the most appropriate reference genes in either mechanically-stimulated cartilage explants or chondrocytes, the stability of the mRNA expression of each reference gene was statistically analysed using four different softwares including the comparative delta-C_t_ method^6^, geNorm^7^, NormFinder^8^, and BestKeeper^9^ which were located on a web-based tool called RefFinder (http://www.leonxie.com). RefFinder integrates and compares the analyses performed by the individual software packages to rank the tested candidate reference genes. Based on these rankings, RefFinder assigns an appropriate weight to an individual reference gene and calculates the geometric mean of their weights for the overall final ranking.

### Statistical analysis

Where appropriate, data are presented as mean ± 95% confidence interval where *N* = 3 (8 MPa data) or 6 explants (all remaining explant data) obtained from the legs of either three or six animals respectively. For primary chondrocytes, *n* = 3 wells per experiment originating from the same pool of cells and were isolated from the legs of three animals; two independent repeat experiments were performed on pools of cells derived from different animals to confirm observed responses on reference gene stability. MMP3 and ACAN data are presented as fold change in expression in the cartilage explants normalised to selected reference genes, as indicated in the text, and relative to the unloaded cDNA samples. MMP3 and ACAN qPCR data were tested for normality (Anderson–Darling) and equal variance (Levene's test) prior to a Student's *t*-test (Minitab 16); where data did not exhibit a normal distribution, a Mann–Whitney test was performed. Differences were considered significant at *P* < 0.05.

## Results

### Reference gene expression levels in cartilage explants and primary chondrocytes

#### Explants

**Fig. 1:**
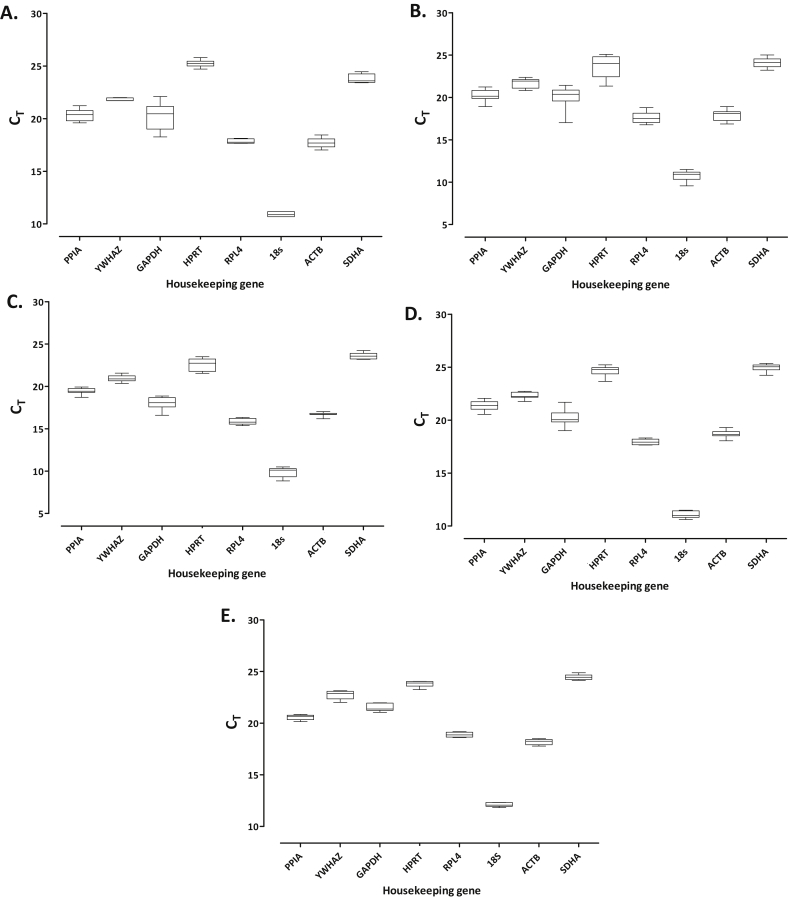
Expression levels of commonly used reference genes for mRNA normalisation in articular cartilage explants subjected to a loading regime of: **[A]** 8 MPa (1 Hz, 15 min; *N* = 3), **[B]** 5 MPa (1 Hz, 15 min; *N* = 6) or **[C]** 2.5 MPa (1 Hz, 15 min; *N* = 6), all of which were analysed for gene expression 24 h post-cessation of load, **[D]** 5 MPa (4 Hz, 15 min; *N* = 6) and gene expression ascertained immediately post-loading, or **[E]** high-density primary articular chondrocytes subjected to tensile strain (7.5% elongation, 1 Hz, 30 min; *N* = 2) and gene expression determined 4 h post-cessation of strain. The box and whisker diagrams illustrate the threshold cycles (C_T_) obtained by qPCR using SYBR^®^ green chemistry and cDNA prepared with 1 μg total RNA.

Reference gene expression levels were measured in 42 explants comprising four different loading conditions (altered magnitude and frequency of load and period post-cessation of load); unloaded explants served as controls. Reference gene expression levels in cartilage explants comprised a mean C_t_ value of 11 for 18s extending to C_t_ values of approximately 24 for HPRT and SDHA [Fig. 1(A)–(D)].

#### Primary chondrocytes

Comparable trends in reference gene expression levels were observed in the isolated primary chondrocytes [Fig. 1(E)]. 18s levels were the most abundant (mean C_t_ of 12), whilst HPRT and SDHA expression levels were the lowest (mean C_t_ of 24).

### Reference gene stability in mechanically-stimulated articular cartilage explants and chondrocytes

To ascertain variation in reference gene expression levels in mechanically-stimulated material, four different software packages, including the comparative delta-C_t_ method, NormFinder, geNorm and BestKeeper, were utilised.

#### Comparative delta-C_t_ method

**Fig. 2:**
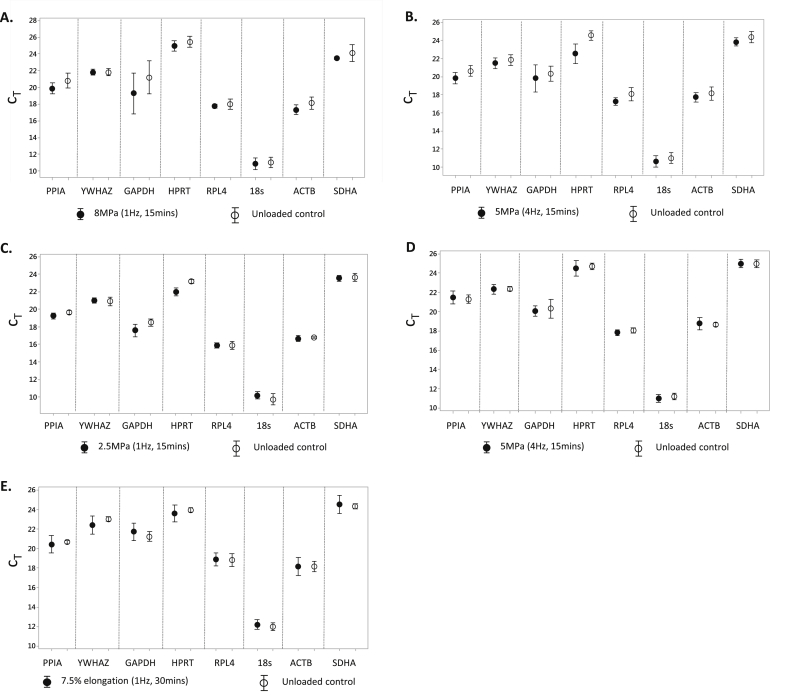
Assessment of gene stability using the comparative delta-C_t_ method of eight commonly used reference genes in articular cartilage explants either left unloaded or subjected to a loading regime of: **[A]** 8 MPa (1 Hz, 15 min; *N* = 3), **[B]** 5 MPa (1 Hz, 15 min; *N* = 6) or **[C]** 2.5 MPa (1 Hz, 15 min; *N* = 6), all of which were analysed for gene expression 24 h post-cessation of load, **[D]** 5 MPa (4 Hz, 15 min; *N* = 6) and gene expression ascertained immediately post-loading, or **[E]** high-density primary articular chondrocytes subjected to tensile strain (7.5% elongation, 1 Hz, 30 min; *N* = 2) and gene expression determined 4 h post-cessation of strain. Data is presented as mean threshold cycle (C_T_) ± standard deviation.

The comparative delta-C_t_ method compares the standard deviation of the C_t_ across experimental samples. To ascertain reference gene stability, loading regimes were analysed independently to achieve a true reflection of effect (Fig. 2). Most notable was the observation that some of the genes, previously surmised as suitable reference genes, were subject to mechano-regulation in cartilage; altering the magnitude of load led to differences in the C_t_ values measured. A large variation in the C_t_ values were observed for GAPDH, both in response to load or explants left unloaded under comparable conditions [Fig. 2(A)–(D)]. In addition, there was variability in PPIA, HPRT and ACTB transcript levels. YWHAZ and RPL4 were the most consistent in response to higher magnitudes of load [8 MPa, 1 Hz; Fig. 2(A)], YWHAZ and 18s in response to a 5 MPa (4 Hz) load [Fig. 2(B)] and YWHAZ, RPL4, ACTB and SDHA in response to a 2.5 MPa (1 Hz) load [Fig. 2(C)]. Interestingly, the time frame at which the experiment was terminated also influenced reference gene transcription. Gene expression was less variable immediately post-cessation of load [Fig. 2(D)], in comparison to transcript levels 24 h post-cessation of load [Fig. 2(A)–(C)], indicating ‘time’ is another influence to be considered; this observation of greater reference gene instability at 24 h compared to directly post-load may reflect the period of time required to detect transcribed *de novo* mechanically-regulated mRNAs. GAPDH gene expression was still identified as being the most variable in explants immediately post-load, along with HPRT and ACTB transcript levels [Fig. 2(D)]. In comparison to the other data sets, very little variation was observed in baseline expression levels in the unloaded explants. Under these experimental loading conditions, YWHAZ, 18s, SDHA and RPL4 were all deemed suitable references genes for use [Fig. 2(D)]. Generally, reference gene expression levels were more variable in the primary cells processed 4 h post-cessation of load compared to their unloaded counterparts [Fig. 2(E)]. Of the eight reference genes measured, 18s and RPL4 were deemed the most stable under these experimental conditions using the comparative delta-C_t_ method.

#### NormFinder

**Fig. 3:**
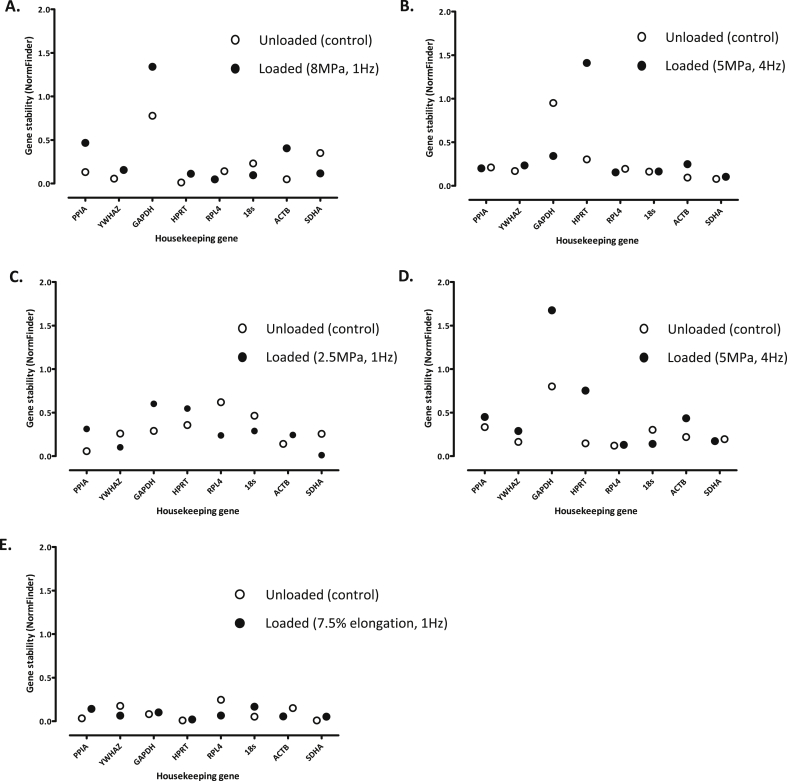
Evaluation of reference gene stability using NormFinder in articular cartilage explants either left unloaded or subjected to a loading regime of: **[A]** 8 MPa (1 Hz, 15 min; *N* = 3), **[B]** 5 MPa (1 Hz, 15 min; *N* = 6) or **[C]** 2.5 MPa (1 Hz, 15 min; *N* = 6), all of which were analysed for gene expression 24 h post-cessation of load, **[D]** 5 MPa (4 Hz, 15 min; *N* = 6) and gene expression ascertained immediately post-loading, or **[E]** high-density primary articular chondrocytes subjected to tensile strain (7.5% elongation, 1 Hz, 30 min; *N* = 2) and gene expression determined 4 h post-cessation of strain.

NormFinder software takes into consideration experimental design and variation between and within groups to assess gene stability^8^. NormFinder also indicated that expression of several of the reference genes altered in cartilage explants under the experimental parameters tested (Fig. 3). At the higher loading magnitude (8 MPa, 1 Hz), GAPDH, PPIA and ACTB were very disparate in terms of gene stability, whereas YWHAZ, HPRT, RPL4 and 18s were more consistent [Fig. 3(A)]. In response to a 5 MPa (4 Hz) load, most of the reference genes measured were considered relatively stable in expression with the least stable being HPRT and GAPDH [Fig. 3(B)]. In contrast, RPL4 and GAPDH were the least stable in expression in explants subjected to a 2.5 MPa (1 Hz) load [Fig. 3(C)], with ACTB transcription the least affected by this regime. With cartilage explants, gene expression analysed directly post-load (5 MPa, 4 Hz) revealed GAPDH and HPRT to be least stable, whereas there was a high degree of stability in the expression of SDHA and RPL4 [Fig. 3(D)]. In contrast, gene stability was found to be more consistent between primary chondrocytes either subjected to tensile strain (7.5%, 1 Hz) or left unstrained, although HPRT and GAPDH were selected as exhibiting the most stable expression and RPL4 conferred least stability [Fig. 3(E)].

#### geNorm

**Fig. 4:**
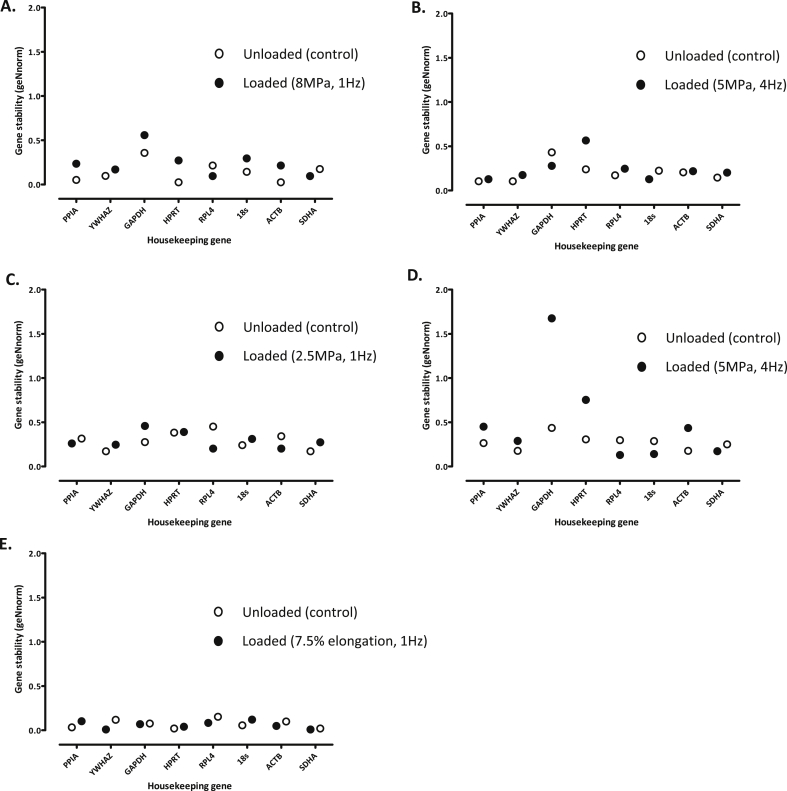
Evaluation of reference gene stability using geNorm in articular cartilage explants either left unloaded or subjected to a loading regime of: **[A]** 8 MPa (1 Hz, 15 min; *N* = 3), **[B]** 5 MPa (1 Hz, 15 min; *N* = 6) or **[C]** 2.5 MPa (1 Hz, 15 min; *N* = 6), all of which were analysed for gene expression 24 h post-cessation of load, **[D]** 5 MPa (4 Hz, 15 min; *N* = 6) and gene expression ascertained immediately post-loading, or **[E]** high-density primary articular chondrocytes subjected to tensile strain (7.5% elongation, 1 Hz, 30 min; *N* = 2) and gene expression determined 4 h post-cessation of strain.

geNorm determines the stability of reference genes by calculating average pair-wise variation between a single reference gene and the other group of reference genes^7^. Interestingly, geNorm identified SDHA, RPL4 and YWHAZ as having the most stable expression at the higher loading magnitude (8 MPa, 1 Hz), whereas there was increased variation in stability of several of the other genes analysed [Fig. 4(A)]. PPIA, ACTB and SDHA transcript levels remained comparable in explants exposed to a 5 MPa (4 Hz) load, with GAPDH and HPRT most variable [Fig. 4(B)]. In contrast, HPRT expression was considered most stable at the lower load of 2.5 MPa (1 Hz), with GAPDH, RPL4 and ACTB levels subject to variation [Fig. 4(C)]. As observed using NormFinder [Fig. 3(D)], geNorm also identified both GAPDH and HPRT as the least stable reference genes directly post-cessation of load [Fig. 4(D)]. geNorm analysis of the primary chondrocytes indicated that SDHA, HPRT and GAPDH were most consistent, with increased variation in the remaining reference genes analysed [Fig. 4(E)].

#### BestKeeper

In comparison to the other software packages, BestKeeper evaluates the inter-gene relationship amongst the tested reference genes to assess mRNA stability^9^; a low correlation between a pair of reference genes likely indicates that one of them was modulated by the experimental condition. BestKeeper demonstrated the least cohesiveness in identifying appropriate reference genes (Supplemental Tables 1–5), identifying inter-gene relationships between several reference genes, particularly in explants subjected to different loading regimens, including HPRT and GAPDH. In contrast, only YWHAZ transcript levels were variable in primary chondrocytes exposed to elongation (Supplemental Table 5).

#### RefFinder

**Fig. 5:**
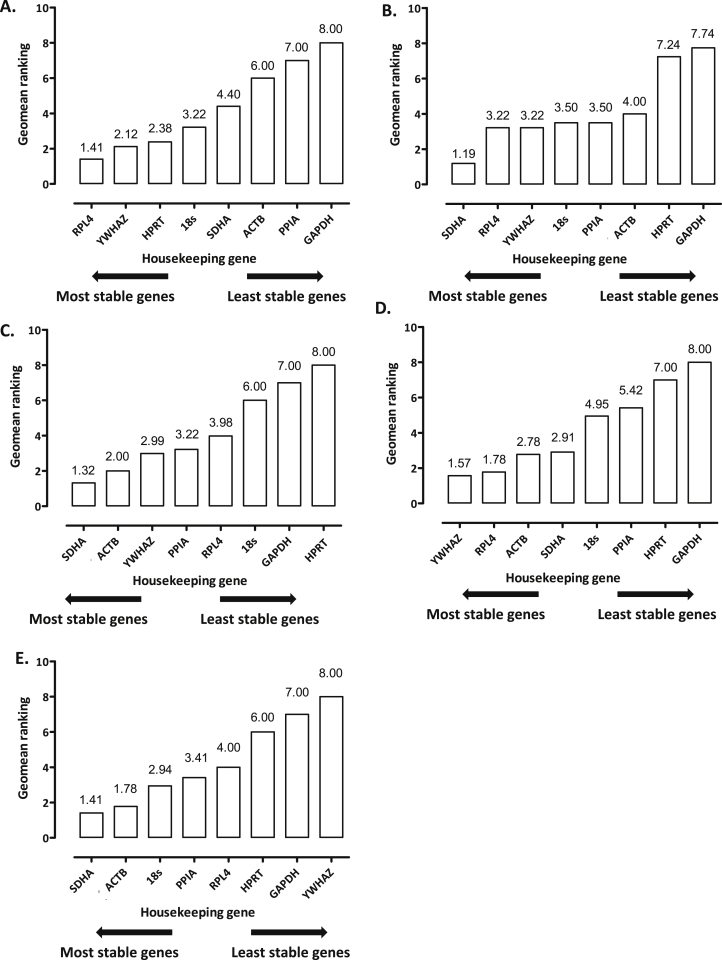
Evaluation of reference gene stability using RefFinder (software that generates its own ranking based on the geometric means of the individual reference genes, having integrated and compared the analyses performed by the individual software packages) in articular cartilage either left unloaded or subjected to a loading regime of: **[A]** 8 MPa (1 Hz, 15 min; *N* = 3), **[B]** 5 MPa (1 Hz, 15 min; *N* = 6) or **[C]** 2.5 MPa (1 Hz, 15 min; *N* = 6), all of which were analysed for gene expression 24 h post-cessation of load, **[D]** 5 MPa (4 Hz, 15 min; *N* = 6) and gene expression ascertained immediately post-loading, or **[E]** high-density primary articular chondrocytes subjected to tensile strain (7.5% elongation, 1 Hz, 30 min; *N* = 2) and gene expression determined 4 h post-cessation of strain.

RefFinder generates its own ranking based on the geometric means of the individual reference genes. When all of the geometric means had been calculated, the reference genes SDHA, YWHAZ or RPL4 were generally identified as having the most stable expression irrespective of loading regime, when the tissue was analysed 24 h post-cessation of load [Fig. 5(A)–(C)]. In most instances, HPRT and GAPDH were found to have the least stable expression, and therefore least appropriate for use. However, at the highest magnitude of load explored in this study (8 MPa, 1 Hz), RefFinder identified HPRT as being one of the most consistent in expression [Fig. 5(A)]. Interestingly, RefFinder indicated that RPL4, along with YWHAZ, ACTB and SDHA were also the most suitable in the experimental set-up where gene expression was analysed immediately post-cessation of load [Fig. 5(D)], and consistent with findings from the other regimens HPRT and GAPDH were least constant in expression. Although SDHA, ACTB and 18s transcript levels were most stable in primary chondrocytes exposed to elongation, YWHAZ and GAPDH were found to be the least appropriate reference genes for use [Fig. 5(E)].

### Impact of reference gene choice on the expression of target genes of interest

**Fig. 6:**
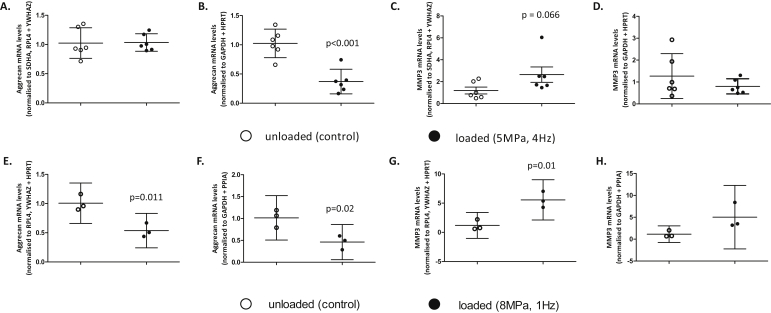
Effect of reference gene selection on ACAN and MMP3 mRNA levels in cartilage explants either subjected to a **[A–D]** 5 MPa load (4 Hz, 15 min; *N* = 6, analysed 24 h post-cessation of load) or a **[E–H]** 8 MPa load (1 Hz, 15 min; *N* = 3, analysed 24 h post-cessation of load) using either SDHA, RPL4 and YWHAZ [A, C] or HPRT, RPL4 and YWHAZ [E, G] identified as most stable, or GAPDH and HPRT [B, D] or GAPDH and PPIA [F, H], identified as the least stable reference genes. Data is presented as mean relative fold change ±95% confidence interval normalised to the indicated reference genes and further normalised to the unloaded control explants; statistical analysis was performed using the Student's *t*-test or Mann–Whitney non-parametric test and statistical significance is indicated.

To assess the importance of selecting the most appropriate reference gene(s) to use, transcript levels of two genes of interest i.e., ACAN and MMP3 were examined in two independent experimental set-ups. Using RefFinder recommendations [Fig. 5(B)], load-induced transcriptional responses in cartilage explants analysed 24 h post-cessation of load were quantified (Fig. 6) using the stable reference genes SDHA, RPL4 and YWHAZ (5 MPa, 4 Hz) and RPL4, YWHAZ and HPRT (8 MPa, 1 Hz) or the least stable reference genes GAPDH and HPRT (5 MPa, 4 Hz) and GAPDH and PPIA (8 MPa, 1 Hz). When normalised to stable reference genes no difference was observed in ACAN mRNA levels (*P* = 0.934; Fig. 6(A)), however when normalised to GAPDH and HPRT, a load-induced 2.8-fold reduction in ACAN transcription was observed [*P* < 0.001; Fig. 6(B)]. Furthermore, MMP3 levels were increased by load when normalised to the stable reference genes [2.4-fold, *P* = 0.066; Mann–Whitney test; Fig. 6(C)], whereas there was no effect when related to the less stable GAPDH and HPRT (*P* = 0.575; Fig. 6(D)). Assessment of cartilage subjected to an 8 MPa load yielded similar effects depending on reference gene selection [Fig. 6(E)–(H)]. ACAN transcription was significantly reduced in response to an 8 MPa load [2-fold, *P* = 0.011; Fig. 6(E)] when normalised to the stable reference genes [Fig. 5(A)]. Similar trends were observed when ACAN data was normalised to the unstable reference genes, however the *P* value denoting significance was not as great [2-fold, *P* = 0.02; Fig. 6(F)]. Furthermore, MMP3 mRNA levels were sensitive to the 8 MPa loading regime (5.4-fold, *P* = 0.01) when normalised to HPRT, RPL4 and YWHAZ [Fig. 6(G)]. In contrast, load-induced expression of MMP3 was not significant when normalised to the unstable reference genes [*P* = 0.09; Fig. 6(H)], even though a 4.5-fold increase in MMP3 transcript levels was observed, due to large sample variability. Data illustrating that comparable trends were observed when ACAN and MMP3 transcript levels were normalised to each independent reference gene in response to an 8 MPa load are also presented for comparison (Supplemental Fig. 1).

## Discussion

In this study, stability of reference gene expression was assessed in two loading models: *ex vivo* cartilage explants subjected to different compressive loading regimes, and secondly, high-density chondrocytes subjected to tensile strain. Both experimental models are routinely used worldwide to identify mechano-signalling events in cartilage chondrocytes in *in vitro* systems. However, to date, only one study has previously been performed to identify stable reference gene(s) for normalisation of transcript levels in isolated articular chondrocytes subjected to load^3^; in this study, of the four genes analysed in cartilage (18s, β-actin, GAPDH and β2-microglobulin), 18s was deemed to have the most stable expression under the experimental conditions tested. Validation of suitable reference genes in mechanically loaded *ex vivo* articular cartilage tissue has not been reported to date, therefore evaluation of reference gene stability is critical to ensure appropriate normalisation of mechanically-regulated target genes of interest. To ascertain whether the same cohort of reference genes exhibit stable expression in native cartilage tissue and isolated chondrocytes subjected to load, both model systems were studied.

To evaluate the stability of candidate reference gene expression in mechanically-stimulated articular cartilage explants and isolated chondrocytes for qPCR normalisation, four commonly-used algorithms e.g., comparative delta-C_t_ method^6^, geNorm^7^, NormFinder^8^ and BestKeeper^9^ were compared. RefFinder was then applied to generate its own ranking based on the geometric means of the individual reference genes, having integrated and compared the analyses performed by the individual software packages. Overall, all of the reference genes analysed were expressed in both cartilage explants and primary chondrocytes with 18s exhibiting the most abundant expression. Analysis of the standard deviations across these reference genes (comparative delta-C_t_ method) demonstrated that the stability of several ‘reference genes’ were affected by the loading regimens utilised.

Irrespective of the software package used for the analysis, HPRT and GAPDH were consistently identified as the least stable reference genes in both cartilage explant and chondrocyte model systems. As GAPDH is an enzyme involved in glycolysis it is not surprising that its transcription alters in response to mechanical load, which places further metabolic demands on the tissue/cells. HPRT is involved in generating purine nucleotides; although transcript levels remained relatively stable in the unloaded specimens, HPRT was observed to be influenced by mechanical load. The only exception to this was the observation of HPRT transcript stability in response to the higher load (8 MPa, 1 Hz), although it is unclear why this may be the case. These findings corroborate other studies demonstrating significant variability in both GAPDH and HPRT expression levels in human^25^ and canine^26^ osteoarthritic articular cartilage, and in chondrocytes exposed to hypoxia^27^. However, specimen source (tissue, primary cell or cell line), developmental stage and stimulus under investigation all need to be taken into consideration when identifying appropriate reference genes for experimental study.

Two of the least stable reference genes identified in our study i.e., GAPDH and HPRT were revealed to be stable in C-28/12 chondrocytes exposed to chondro-protective agents^28^. Furthermore, HPRT and PPIA have previously been identified as candidate reference genes in the ATDC5 chondroprogenitor cell line^21^. In an *in vivo* model of inflammatory joint pathology (K/BxN), GAPDH transcript levels were significantly reduced whereas HPRT, along with β_2_-microglobulin and RPL13a were validated as most constant in expression^29^, again highlighting how the model system impacts on the selection of reference gene(s) used.

Only one study has reported on reference gene suitability in cartilage mechanobiology^3^, and very few studies have investigated the validity of reference gene stability in response to mechanical load in other tissues. Yurube *et al.* utilised a rat tail compression loading-induced disc degeneration model and identified GAPDH and ACTB as most and least stable in expression, respectively^20^. Analysis of individual cell populations of the lung to cyclic mechanical strain (30% elongation, 2–6 h) indicated that stability varied between cell type and loading duration^30^. In our study, the stability of reference genes in explants was not significantly altered by delaying the extraction of RNA post-load, validating their use as appropriate reference genes. Although the period of time required for transcriptional regulation and processing could influence this outcome, the fact that the most and least stable reference genes were generally consistent over time suggests that the effects observed reflect true gene stability or instability and not differences in transcriptional processing times. Pinhu *et al.* concluded that at least four reference genes should be selected^30^, reflecting suggestions proposed by the MIQE guidelines which recommend the use of at least three^4^, ^5^.

Clearly, reference gene choice can strongly influence outcome, with observable under and over-estimations of fold changes in transcript levels when using unstable reference genes for normalisation. As evidenced in this study, use of inappropriate reference genes suggested a significant load-induced reduction in ACAN transcription, but when normalised to the three most stable reference genes, ACAN expression levels were unaltered by this particular regime (5 MPa, 4 Hz). However, when higher magnitudes of load were applied to the cartilage (8 MPa, 1 Hz), a significant reduction in ACAN and induction of MMP3 transcription (stable reference genes) were either significant to a lesser extent or not significant respectively, when utilising the less stable GAPDH and PPIA, therefore the data is potentially subject to incorrect interpretation.

This study defines the necessary requirements to achieve accurate normalisation of gene expression in mechanical experiments on *ex vivo* articular cartilage and high-density articular chondrocytes, and provides a conduit for researchers to ascertain the most appropriate reference gene(s) for use in their *in vitro* mechanobiology studies following the recommendations suggested in the MIQE guidelines^4^, ^5^. Furthermore, all four commonly used algorithms for reference gene selection identified similar genes whose expression remained unaffected by mechanical load and those that were inherently unstable, indicating that any combination of these software tools are suitable for use. In conclusion, this study has demonstrated that many common ‘reference genes’ may not be suitable, depending on the model system, for *in vitro* cartilage chondrocyte mechanobiology studies and reinforces the principle that careful validation of reference genes is essential for each independent experiment to obtain robust and reproducible qPCR data for analysis and interpretation.

## Contributions

AAS and PS contributed to data acquisition, data analysis and interpretation, statistical analysis and critical revision of the draft, SJG contributed to study conception and design, data acquisition, data analysis and interpretation and critical revision of the draft, VCD contributed to data analysis and interpretation, critical revision of the draft and obtaining funding, EJB contributed to study conception and design, data analysis and interpretation, statistical analysis, drafting the article and obtaining funding. All authors approved the final manuscript and take full responsibility for the integrity of the study.

## Competing interests

The authors have no conflicts of interest.

## Role of the funding source

The sponsors had no involvement in the study.

## Figures and Tables

**Table I:** Primer sequences, mRNA accession numbers and amplicon sizes of all primer pairs used for qPCR analyses

Gene symbol	Accession number	Forward primer (5′–3′)	Reverse primer (5′–3′)	Amplicon size (bp)	Ref
β-act	NM_173979	CATCGCGGACAGGATGCAGAAA	CCTGCTTGCTGATCCACATCTGCT	157	18
GAPDH	NM_001034034	TTGTCTCCTGCGACTTCAACAGCG	CACCACCCTGTTGCTGTAGCCAAAT	133	17
SDHA	NM_175814	GATGTGGGATCTAGGAAAAGGCCTG	ACATGGCTGCCAGCCCTACAGA	104	18
18s	NR_036642	GCAATTATTCCCCATGAACG	GCCTCACTAAACCATCCAA	123	16
RPL4	NM_001014894	TTTGAAACTTGCTCCTGGTGGTCAC	TCGGAGTGCTCTTTGGATTTCTGG	199	18
YWHAZ	NM_174814	CTGAGGTTGCAGCTGGTGATGACA	AGCAGGCTTTCTCAGGGGAGTTCA	180	18
HPRT	NM_001034035	TAATTATGGACAGGACCGAACGGCT	TTGATGTAATCCAACAGGTCGGCA	127	18
PPIA	NM_178320	GGTGGTGACTTCACACGCCATAATG	CTTGCCATCCAACCACTCAGTCTTG	186	18
ACAN	NM_173981	GCTACCCTGACCCTTCATC	AAGCTTTCTGGGATGTCCAC	76	17
MMP3	XM_586521	TGGAGATGCTCACTTTGATGATG	GAGACCCGTACAGGAACTGAATG	221	19

## Appendix A Supplementary data

Table 1. Pair-wise correlation analysis of candidate housekeeping genes using BestKeeper to evaluate inter-gene relationships amongst the tested reference genes in articular cartilage either left unloaded or subjected to a loading regime of 8 MPa (1 Hz, 15 min; analysed 24 h post-cessation of load). Correlation coefficients (r) and statistical significance (*P* values) are presented for each comparison (*N* = 3).

Table 2. Pair-wise correlation analysis of candidate housekeeping genes using BestKeeper to evaluate inter-gene relationships amongst the tested reference genes in articular cartilage either left unloaded or subjected to a loading regime of 5 MPa (4 Hz, 15 min; analysed 24 h post-cessation of load). Correlation coefficients (r) and statistical significance (*P* values) are presented for each comparison (*N* = 6).

Table 3. Pair-wise correlation analysis of candidate housekeeping genes using BestKeeper to evaluate inter-gene relationships amongst the tested reference genes in articular cartilage either left unloaded or subjected to a loading regime of 2.5 MPa (1 Hz, 15 min; analysed 24 h post-cessation of load). Correlation coefficients (r) and statistical significance (*P* values) are presented for each comparison (*N* = 6).

Table 4. Pair-wise correlation analysis of candidate housekeeping genes using BestKeeper to evaluate inter-gene relationships amongst the tested reference genes in articular cartilage either left unloaded or subjected to a loading regime of 5 MPa (4 Hz, 15 min; analysed directly post-cessation of load). Correlation coefficients (r) and statistical significance (*P* values) are presented for each comparison (*N* = 6).

Table 5. Pair-wise correlation analysis of candidate housekeeping genes using BestKeeper to evaluate inter-gene relationships amongst the tested reference genes in primary articular chondrocytes either left unloaded or subjected to a loading regime of 7.5% elongation (1 Hz, 30 min; analysed 4 h post-cessation of load). Correlation coefficients (r) and statistical significance (*P* values) are presented for each comparison (*N* = 2).

Fig. 1. Effect of reference gene selection on ACAN and MMP3 mRNA levels in cartilage explants subjected to an 8 MPa load (1 Hz, 15 min; *N* = 3, analysed 24 h post-cessation of load) using either HPRT, RPL4 and YWHAZ [A–C, F–H] identified as most stable, or GAPDH and PPIA [D–E, I–J], identified as the least stable reference genes. Data is presented as mean relative fold change ±95% confidence interval normalised to the indicated reference gene and further normalised to the unloaded control explants; statistical analysis was performed using the Student's *t*-test and statistical significance is indicated.
