# Supplementary material for: Importance of reference gene selection for articular cartilage mechanobiology studies
Source: Osteoarthritis Cartilage. 2016 Apr;24(4):719–30. doi: 10.1016/j.joca.2015.11.007 (PMC4819451; doi:10.1016/j.joca.2015.11.007)
Supplement: Supplementary file 1 [file mmc1.pptx]

## Slide 1
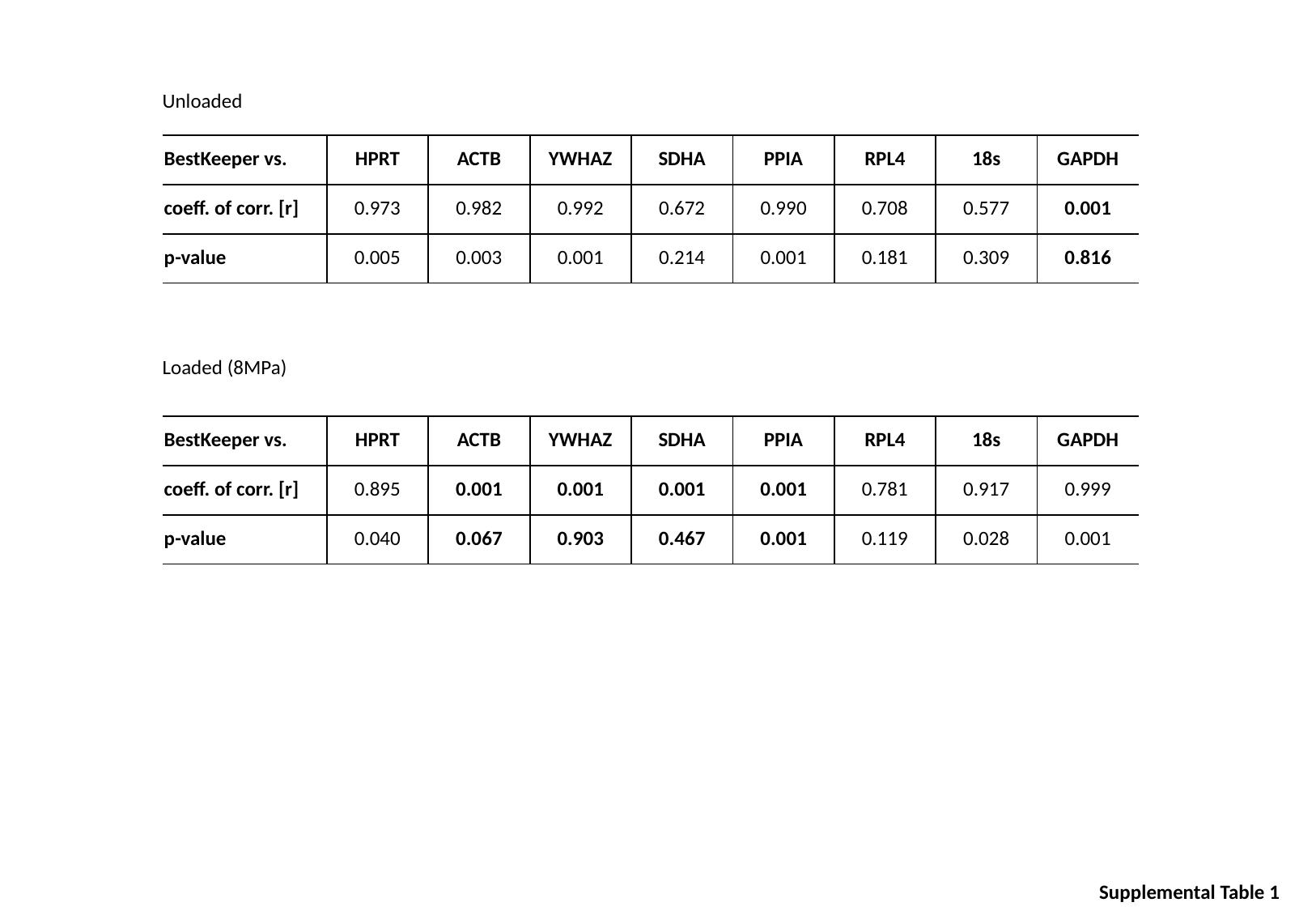

Unloaded
| BestKeeper vs. | HPRT | ACTB | YWHAZ | SDHA | PPIA | RPL4 | 18s | GAPDH |
| --- | --- | --- | --- | --- | --- | --- | --- | --- |
| coeff. of corr. [r] | 0.973 | 0.982 | 0.992 | 0.672 | 0.990 | 0.708 | 0.577 | 0.001 |
| p-value | 0.005 | 0.003 | 0.001 | 0.214 | 0.001 | 0.181 | 0.309 | 0.816 |
Loaded (8MPa)
| BestKeeper vs. | HPRT | ACTB | YWHAZ | SDHA | PPIA | RPL4 | 18s | GAPDH |
| --- | --- | --- | --- | --- | --- | --- | --- | --- |
| coeff. of corr. [r] | 0.895 | 0.001 | 0.001 | 0.001 | 0.001 | 0.781 | 0.917 | 0.999 |
| p-value | 0.040 | 0.067 | 0.903 | 0.467 | 0.001 | 0.119 | 0.028 | 0.001 |
Supplemental Table 1

## Slide 2
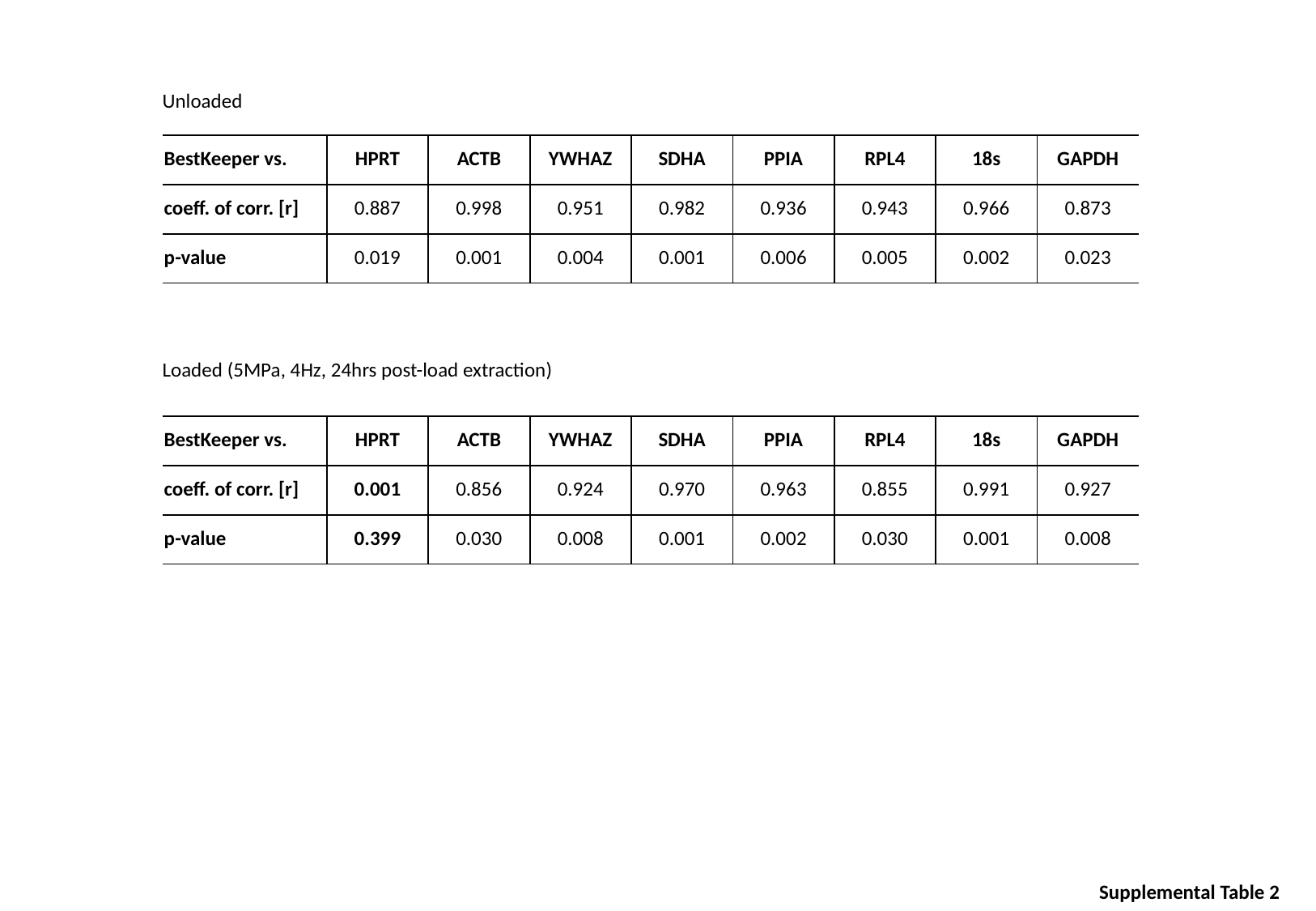

Unloaded
| BestKeeper vs. | HPRT | ACTB | YWHAZ | SDHA | PPIA | RPL4 | 18s | GAPDH |
| --- | --- | --- | --- | --- | --- | --- | --- | --- |
| coeff. of corr. [r] | 0.887 | 0.998 | 0.951 | 0.982 | 0.936 | 0.943 | 0.966 | 0.873 |
| p-value | 0.019 | 0.001 | 0.004 | 0.001 | 0.006 | 0.005 | 0.002 | 0.023 |
Loaded (5MPa, 4Hz, 24hrs post-load extraction)
| BestKeeper vs. | HPRT | ACTB | YWHAZ | SDHA | PPIA | RPL4 | 18s | GAPDH |
| --- | --- | --- | --- | --- | --- | --- | --- | --- |
| coeff. of corr. [r] | 0.001 | 0.856 | 0.924 | 0.970 | 0.963 | 0.855 | 0.991 | 0.927 |
| p-value | 0.399 | 0.030 | 0.008 | 0.001 | 0.002 | 0.030 | 0.001 | 0.008 |
Supplemental Table 2

## Slide 3
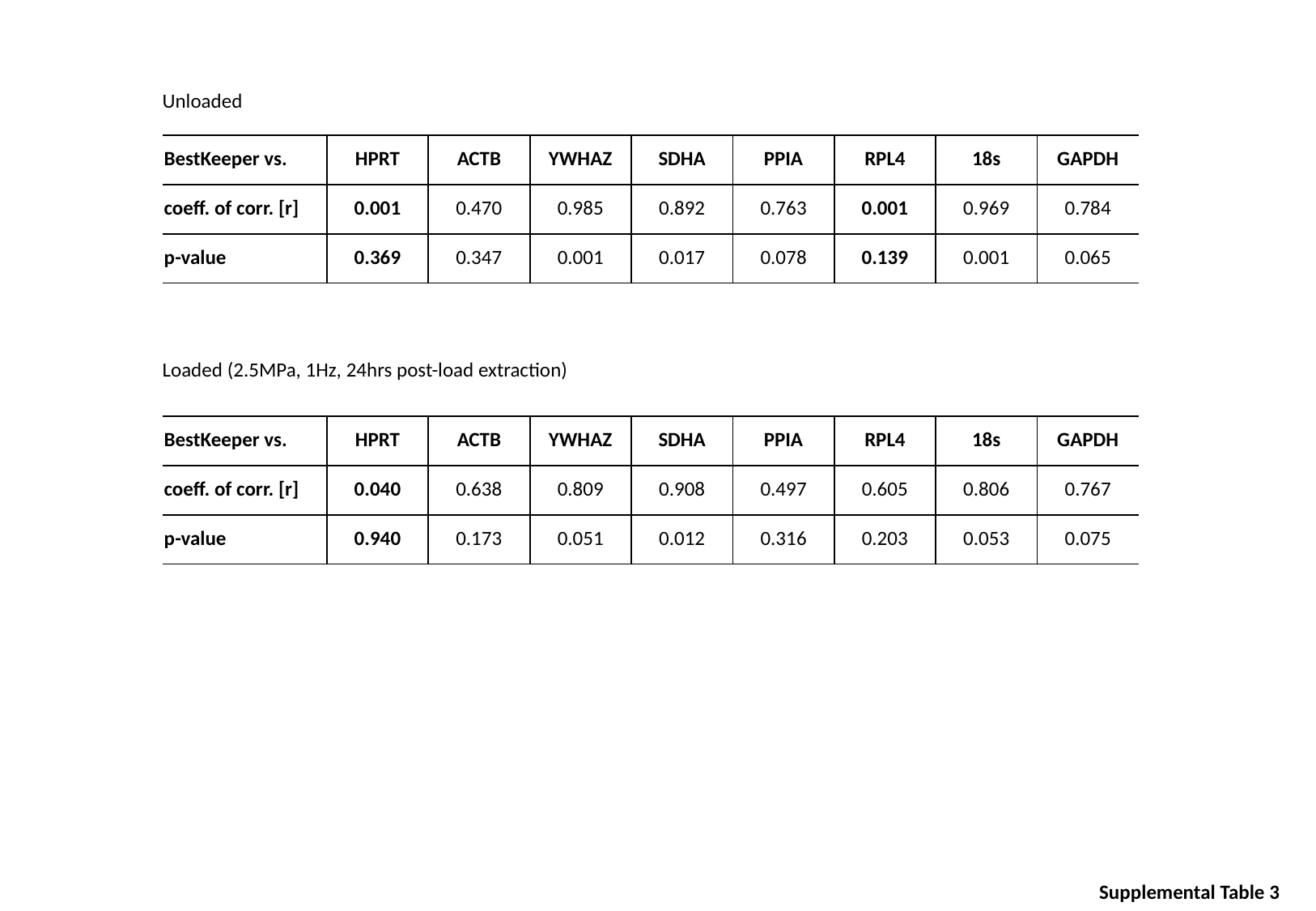

Unloaded
| BestKeeper vs. | HPRT | ACTB | YWHAZ | SDHA | PPIA | RPL4 | 18s | GAPDH |
| --- | --- | --- | --- | --- | --- | --- | --- | --- |
| coeff. of corr. [r] | 0.001 | 0.470 | 0.985 | 0.892 | 0.763 | 0.001 | 0.969 | 0.784 |
| p-value | 0.369 | 0.347 | 0.001 | 0.017 | 0.078 | 0.139 | 0.001 | 0.065 |
Loaded (2.5MPa, 1Hz, 24hrs post-load extraction)
| BestKeeper vs. | HPRT | ACTB | YWHAZ | SDHA | PPIA | RPL4 | 18s | GAPDH |
| --- | --- | --- | --- | --- | --- | --- | --- | --- |
| coeff. of corr. [r] | 0.040 | 0.638 | 0.809 | 0.908 | 0.497 | 0.605 | 0.806 | 0.767 |
| p-value | 0.940 | 0.173 | 0.051 | 0.012 | 0.316 | 0.203 | 0.053 | 0.075 |
Supplemental Table 3

## Slide 4
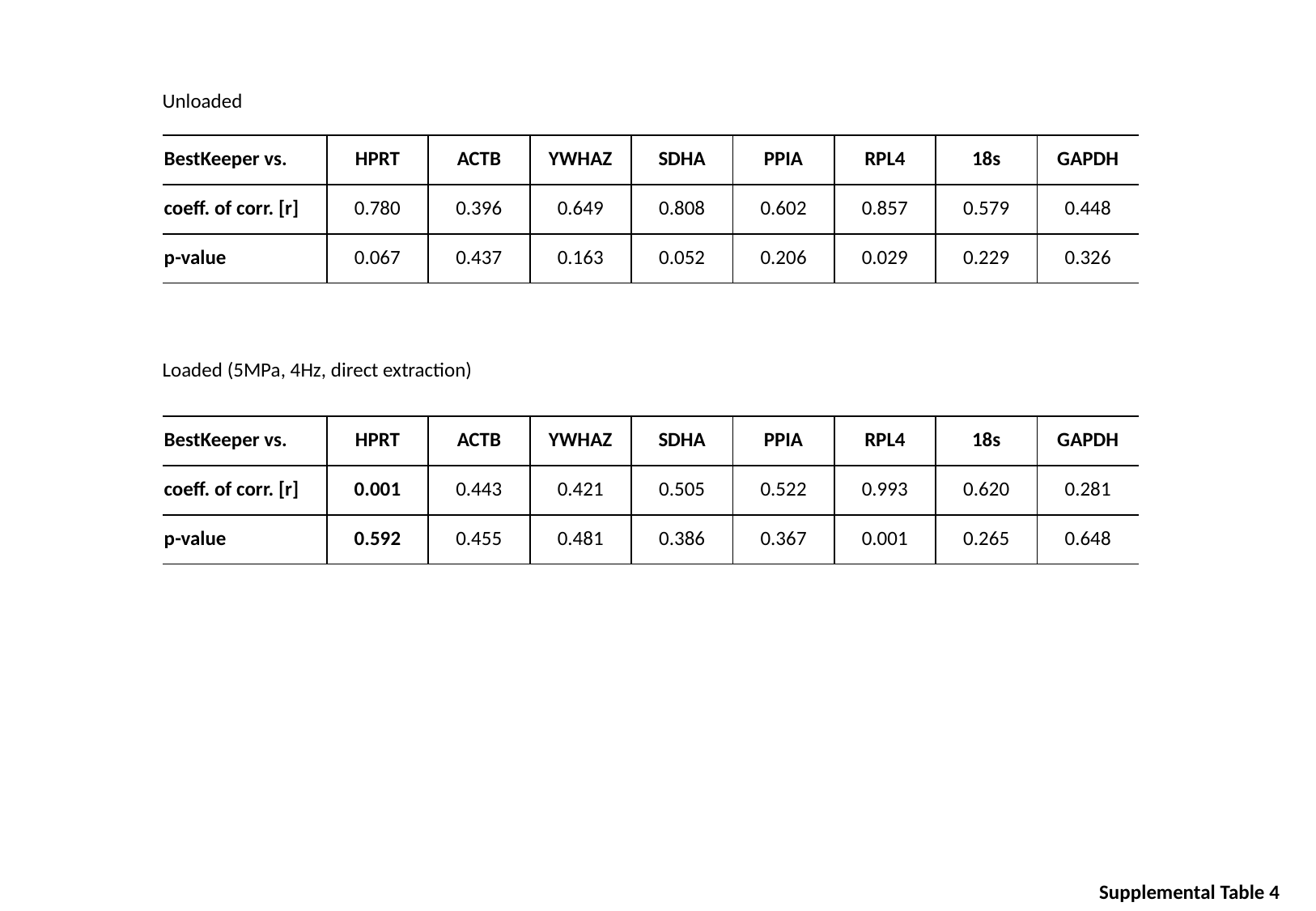

Unloaded
| BestKeeper vs. | HPRT | ACTB | YWHAZ | SDHA | PPIA | RPL4 | 18s | GAPDH |
| --- | --- | --- | --- | --- | --- | --- | --- | --- |
| coeff. of corr. [r] | 0.780 | 0.396 | 0.649 | 0.808 | 0.602 | 0.857 | 0.579 | 0.448 |
| p-value | 0.067 | 0.437 | 0.163 | 0.052 | 0.206 | 0.029 | 0.229 | 0.326 |
Loaded (5MPa, 4Hz, direct extraction)
| BestKeeper vs. | HPRT | ACTB | YWHAZ | SDHA | PPIA | RPL4 | 18s | GAPDH |
| --- | --- | --- | --- | --- | --- | --- | --- | --- |
| coeff. of corr. [r] | 0.001 | 0.443 | 0.421 | 0.505 | 0.522 | 0.993 | 0.620 | 0.281 |
| p-value | 0.592 | 0.455 | 0.481 | 0.386 | 0.367 | 0.001 | 0.265 | 0.648 |
Supplemental Table 4

## Slide 5
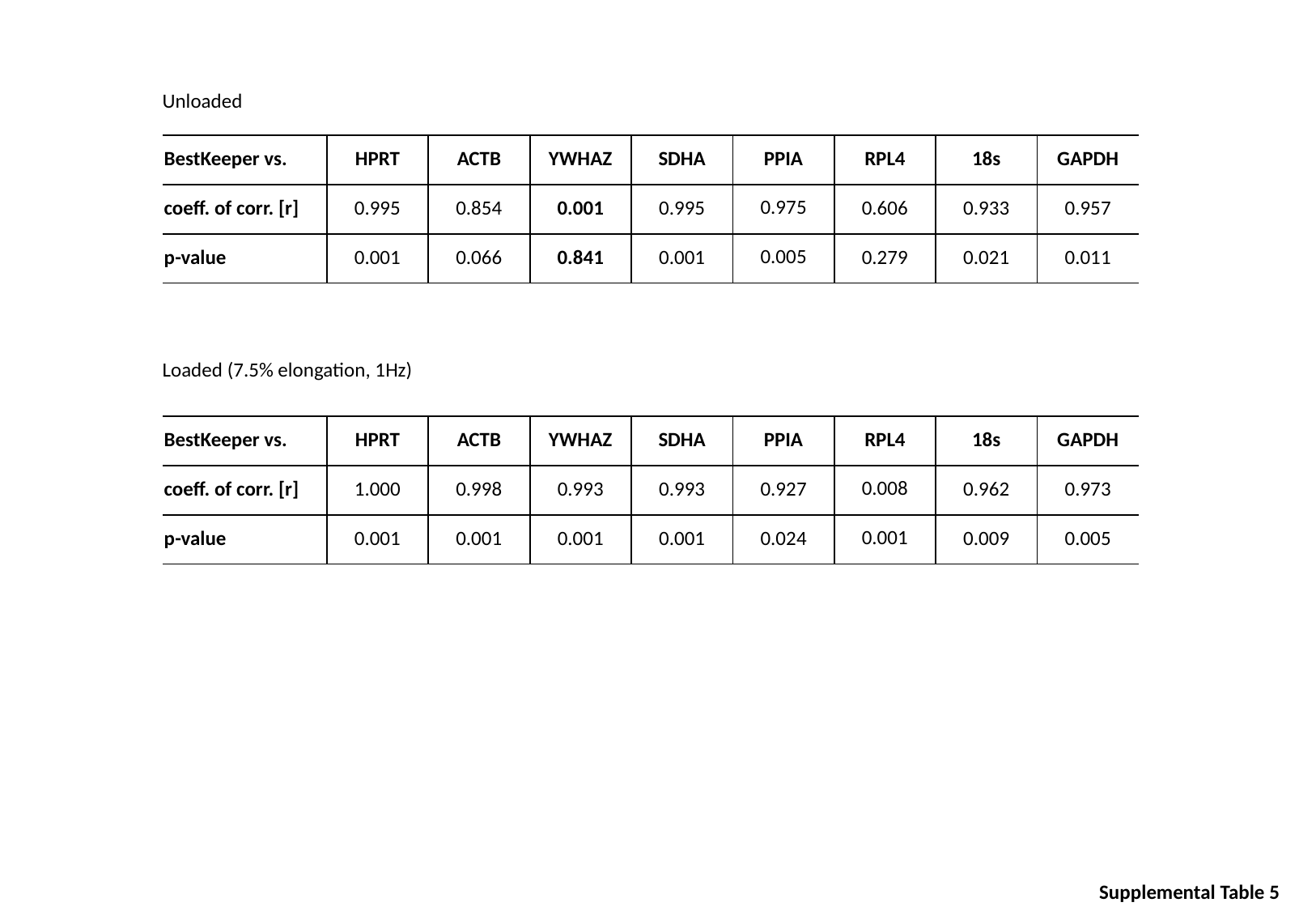

Unloaded
| BestKeeper vs. | HPRT | ACTB | YWHAZ | SDHA | PPIA | RPL4 | 18s | GAPDH |
| --- | --- | --- | --- | --- | --- | --- | --- | --- |
| coeff. of corr. [r] | 0.995 | 0.854 | 0.001 | 0.995 | 0.975 | 0.606 | 0.933 | 0.957 |
| p-value | 0.001 | 0.066 | 0.841 | 0.001 | 0.005 | 0.279 | 0.021 | 0.011 |
Loaded (7.5% elongation, 1Hz)
| BestKeeper vs. | HPRT | ACTB | YWHAZ | SDHA | PPIA | RPL4 | 18s | GAPDH |
| --- | --- | --- | --- | --- | --- | --- | --- | --- |
| coeff. of corr. [r] | 1.000 | 0.998 | 0.993 | 0.993 | 0.927 | 0.008 | 0.962 | 0.973 |
| p-value | 0.001 | 0.001 | 0.001 | 0.001 | 0.024 | 0.001 | 0.009 | 0.005 |
Supplemental Table 5

## Slide 6
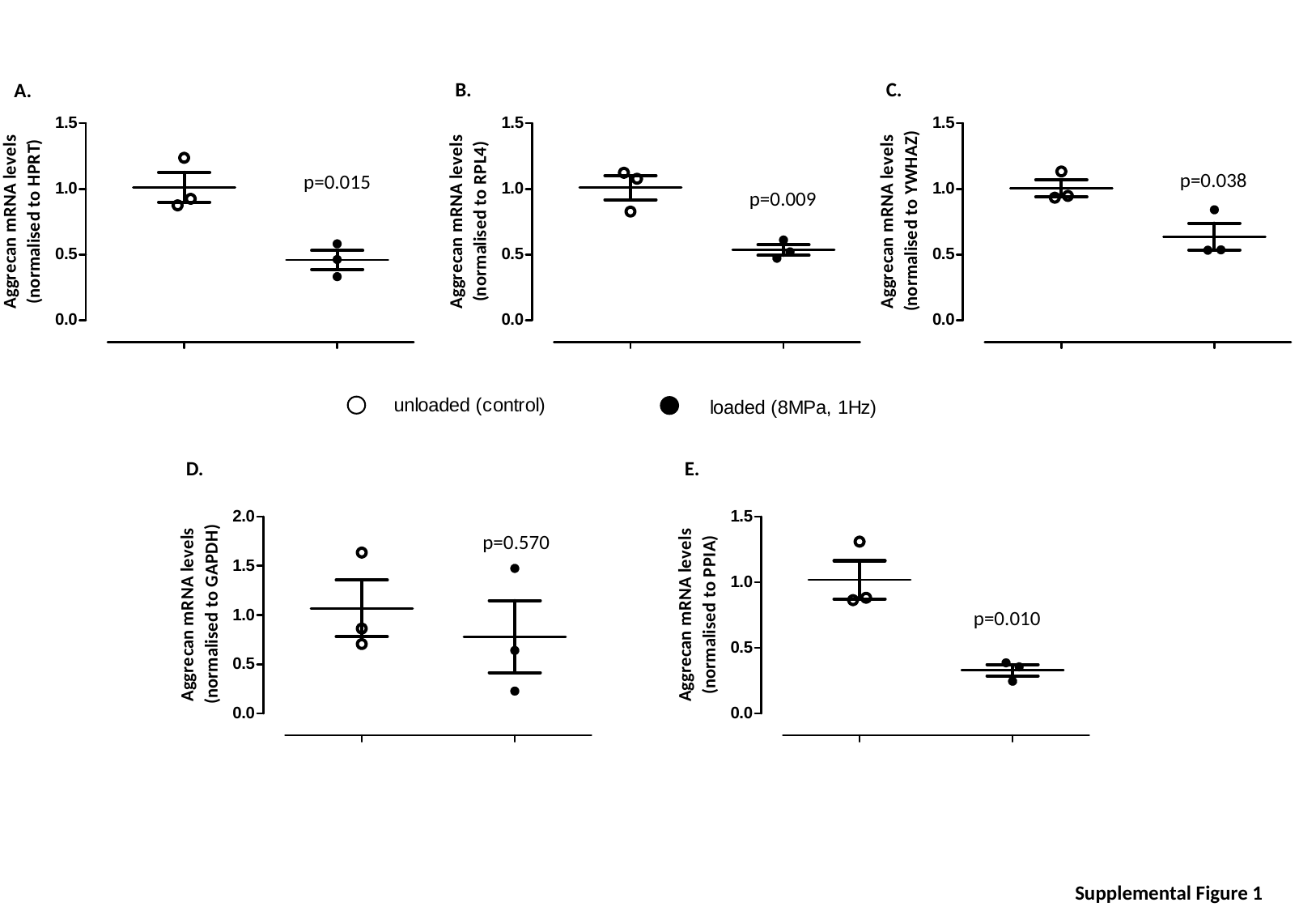

B.
C.
A.
p=0.038
p=0.015
p=0.009
D.
E.
p=0.570
p=0.010
Supplemental Figure 1

## Slide 7
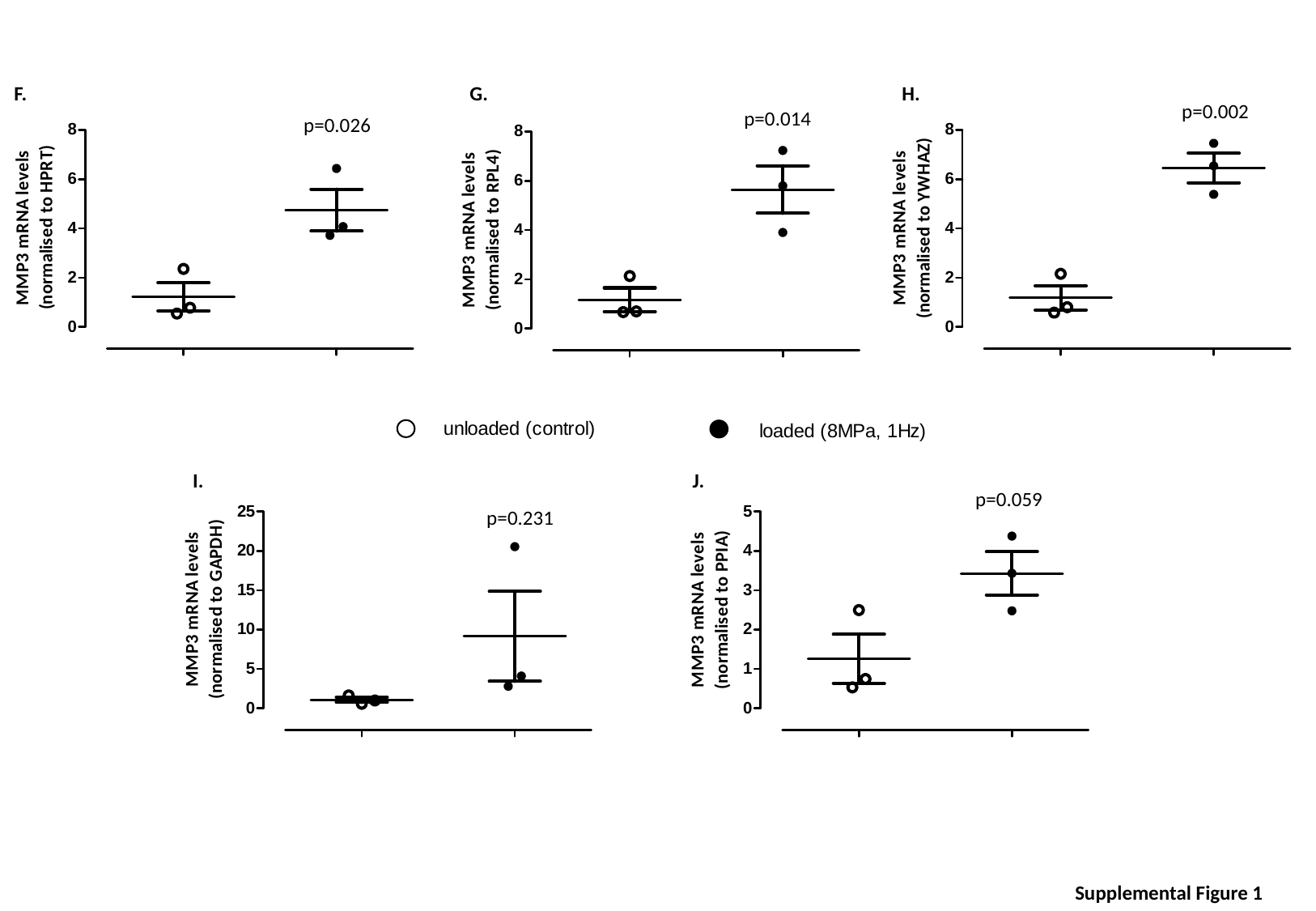

H.
G.
F.
p=0.002
p=0.014
p=0.026
I.
J.
p=0.059
p=0.231
Supplemental Figure 1
